# Failure Analysis in Multiple TKA Revisions—Periprosthetic Infections Remain Surgeons’ Nemesis

**DOI:** 10.3390/jcm11020376

**Published:** 2022-01-13

**Authors:** Stephanie Kirschbaum, Sarah Erhart, Carsten Perka, Robert Hube, Kathi Thiele

**Affiliations:** 1 Centre for Musculoskeletal Surgery, Charité-University Hospital Germany, 10117 Berlin, Germany; saraherhart@gmx.de (S.E.); carsten.perka@charite.de (C.P.); kathi.thiele@charite.de (K.T.); 2 Orthopaedische Chirurgie Muenchen, OCM-Clinic, 81369 Munic, Germany; robert.hube@ocm-muenchen.de

**Keywords:** periprosthetic joint infection, total knee arthroplasty, revision surgery, survivorship, revision knee arthroplasty, multiple revisions

## Abstract

Background: The aim of this study was to categorize reasons for failure and to analyze the survivorship of multiple total knee arthroplasty (TKA) revisions. Methods: The study retrospectively evaluated all multiple TKA revisions performed between 2005 and 2015 at the authors’ institutions. Sixty-three patients (35 female, 28 male, age 64 ± 10 years, follow-up 55 ± 36 months) underwent a total of 157 re-revision TKA surgeries (range 2–5). The revision indications were divided up into main diagnoses. Survivorship was evaluated by mixed model analysis. Results: The main overall reason for re-revision was periprosthetic joint infection (PJI) (48%), followed by instability (12%), polyethylene wear (11%), malpositioning (8%), and aseptic loosening (8%). Survivorship shortened with an increasing number of revision surgeries (*p* = 0.003). While PJI was in 38% of all cases, the reason for the first revision, incidence increased constantly with the number of revisions (48% at second revision, 55% at third revision, 86% at fourth revision, and 100% at fifth revision, *p* = 0.022). If periprosthetic infection caused the first revision, patients showed an average of two more septic revisions at follow-up than patients with an aseptic first revision indication (*p* < 0.001). In 36% of cases, the reason for follow-up surgery in case of periprosthetic infection was again PJI. Conclusion: The probability of survival of the implanted knee arthroplasty is significantly reduced with each subsequent revision. Periprosthetic infection is the main cause of multiple revisions.

## 1. Introduction

The number of total knee arthroplasties (TKA) is continually rising worldwide, and therefore, also the number of revision TKAs. The Swedish knee arthroplasty register showed an increase from 2796 to 39,219 revision cases between 1976 and 2018 [1], resulting in a huge challenge for the future health care system.

Whereas the risk for revision of primary TKA is estimated to be 5% 10 years post-operatively, the risk for re-revision is calculated as 10–14% four to eight years post-operatively [2,3,4].

Therefore, the analysis of TKA failure mechanisms is crucial to improve surgical techniques, proper implant selection, and consequently, TKA survivorship. Periprosthetic joint infection (PJI) (14–38%), instability (8–30%), malposition (3–20%), aseptic loosening (22–41%), polyethylene wear (3–25%), and pain for unknown reasons (6–10%) have already been confirmed as the main causes for the first revision of TKAs in the literature as well as register data [2,5,6,7,8,9]. Mortazavi et al. even described up to 44% failure rate due to PJI as well as a decreased survivorship of revision TKA. Furthermore, 83% of all patients demonstrated early failure within two years [10].

However, while failure analyses of first revisions have been evaluated frequently—especially by the different arthroplasty register data—analyses of multiple revision TKA are hardly represented in the literature [11,12]. As arthroplasty registers are not able to verify and evaluate indications for revision, there is a natural limitation concerning the interpretation of register data. Furthermore, register data is biased due to the use of different PJI criteria and therapy algorithms because mandatory guidelines do not exist. Therefore, single-center studies are crucial to evaluate the indication of TKA re-revision as they are not biased by different diagnostic and treatment algorithms. The aim of this single-center study, in addition to categorizing reasons for failure and analyzing survivorship in multiple revision TKA, is to define TKA revision indications that have a high risk of resulting in re-revisions. In particular, the importance of periprosthetic infections caused by revision surgery and the potential risk for the necessity of multiple revisions shall be characterized in more detail.

## 2. Materials and Methods

**Figure 1:**
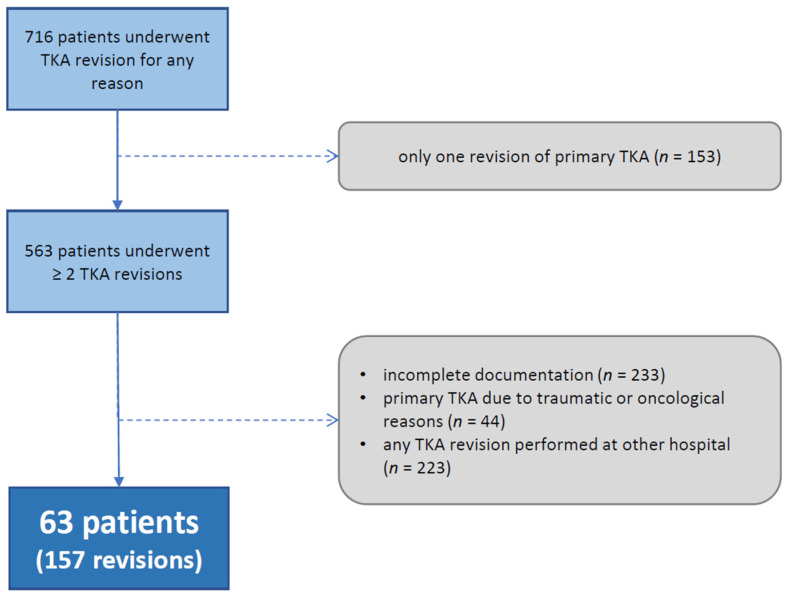
Patient enrollment. TKA = total knee replacement.

The institutional ethics committee approved the study protocol (approval number: EA1/341/15). The institutional database was retrospectively analyzed for patients undergoing TKA revision for any reason at the author’s department between January 2005 and August 2015. Overall, 716 patients were identified and the patients’ histories were checked for exclusion and inclusion criteria. Every surgery, except for primary TKA, had to be performed at the author’s institution to avoid bias caused by different diagnostic and treatment algorithms. If any of the TKA revisions were performed at another hospital (*n* = 456), the patient was excluded. Patients who underwent only one revision after primary TKA were not included in this evaluation (*n* = 153). If the primary TKA was performed for oncologic or post-traumatic reasons (*n* = 44), the patient was also excluded from analysis. Finally, 63 patients with at least two revisions of primary TKA—all performed at the author’s hospital—were included in the study. Patient enrolment is shown in Figure 1. Altogether, 157 TKA revisions were performed within those 63 patients. Hereby, septic multiple stage revisions caused by infection were considered as one event and were counted as a single operation (Figure 1).

The identified patients were interviewed for further complications or revisions by phone or during annual control appointments at the institution’s outpatient department (=final follow-up). In case of death (*n* = 4), the patients’ relatives gave this information.

Demographic parameters such as gender, age, and body mass index (BMI) were documented. Furthermore, details concerning the revision surgery such as the operated side, number and date of the revisions, used implant design (unicondylar, bicondylar, condylar constraint, rotating, and total hinge design), type of fixation (cemented, cementless, hybrid technique) as well as the reasons for the revision and surgical procedure were collected. The reason for revision was assigned to one of the six main diagnoses such as polyethylene wear, aseptic loosening, instability, periprosthetic infection, malpositioning (including malalignment, malrotation, patellofemoral maltracking) and rare causes of failure (periprosthetic fracture, arthrofibrosis, defect of hinge mechanism) following the diagnostic algorithm described by Thiele et al. [13]. Diagnostics were performed using laboratory tests and plain anteroposterior and lateral radiographs; C-reactive protein (CRP) and full-length standing radiographs were included. All patients with suspected PJI underwent a diagnostic arthrocentesis. The diagnosis of periprosthetic infection was related to the criteria defined by the Musculoskeletal Infection Society and the American Academy of Orthopaedic Surgeons (AAOS) [14].

The diagnosis of aseptic loosening was suggested if a circumferential radiolucency line was detected on the x-ray without being able to find any criteria of PJI. Polyethylene wear was established based on macroscopic and microscopic findings according to type I of the classification system described by Krenn et al. [15]. Failures were assigned to the instability group if patients reported subjective instability and giving way of the joint as well as if clinical and radiological examinations showed anteroposterior or mediolateral instability [16,17]. To rule out mechanical axis misalignment radiographs were used. The diagnosis of malalignment was radiologically defined by a pathological component positioning of >5° varus or valgus in the coronal plane, an anterior slope (<0°) or an excessive posterior slope (>10°) [5]. Patients with patellar maltracking and negative findings on a routine workup underwent CT scanning to exclude malrotation [18]. Malrotation was defined as external femoral rotation of >10° or internal rotation > 5° [19,20].

**Figure 2:**
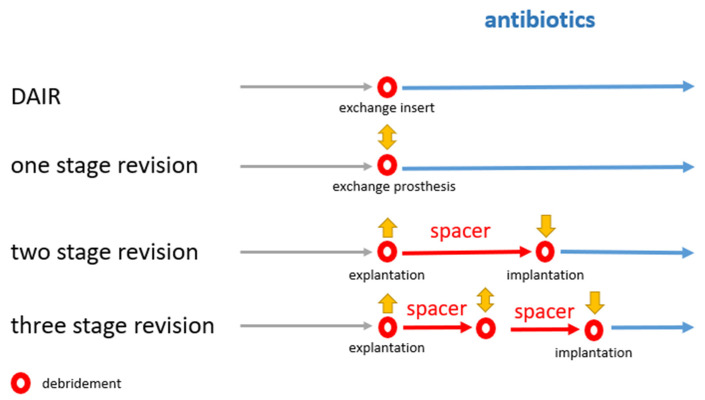
Overview of surgical procedures in case of periprosthetic joint infection as the reason for revision. DAIR = debridement, antibiotics and implant retention.

The survivorship of the prosthesis was defined as the time between two revisions or between the last revision and the time of follow-up. In the case of a septic revision, the subsequent procedure was differentiated into DAIR (debridement, antibiotics, and implant retention), septic one-, two-, or multiple-stage revisions depending on microbiological findings, the health of the patient, and the local wound situation as described by Renz et al. [21] (Figure 2). A one-stage septic revision was performed in the case of chronic prosthesis infection without complicating factors (no preliminary revisions, no fistulae, no difficulty treating detected pathogens).

Statistical analysis: Data was analysed using Statistical Package für Social Sciences. (SPSS, IBM SPSS Statistics Version 25, IBM Corp., Amonk, NY, USA). The data distribution of each metric parameter was checked using the Kolmogorov–Smirnov test. The average and standard deviation of the results were displayed. The influence of demographic parameters (gender, BMI, age) on the number of septic revisions was analysed using the Mann–Whitney U test as the data showed no normal distribution. The comparison of nominal data was performed using the Chi^2^ and Monte Carlo tests. The survivorship of TKA depending on the identified reason for (re-)revision was evaluated using mixed model analysis (“Repeated Covariance Type“). *p* < 0.05 was considered statistically significant.

## 3. Results

### 3.1. Patient Characteristics

Overall, 63 patients were included (28 men (44%) and 35 women (56%)), having at least two revisions. In total, this resulted in a number of 157 revision procedures, which formed the basis for further calculations. The left knee was treated in 27 patients (43%) and the right in 36 (57%). The average age was 63.5 ± 10.4 years when the primary TKA was implanted. The average age at the last revision was 71.8 ± 9.5 years. The average BMI at the first revision was 32 kg/m^2^ ± 7.2. Twenty patients underwent a third revision (32%), seven patients a fourth revision (11%), and four patients had five revisions of their TKA (6%).

### 3.2. Follow-Up

The average time between the last revision and follow-up was 56 months ± 36, between the primary TKA and the last revision was 95 months ± 80, and between the primary TKA and follow-up was 150 months ± 78.

At the final follow-up 89% of all patients (*n* = 56) had no further surgery and in 3% (*n* = 2) an arthrodesis (ex domo) was carried out. Two percent (*n* = 1) underwent an amputation after receiving another arthrodesis ex domo. Six percent (*n* = 4) died.

### 3.3. Reasons for Revision

Overall, the main reason for re-revision was PJI (48%, *n* = 75) followed by instability (12%, *n* = 19) and polyethylene wear (11%, *n* = 17). Malpositioning (*n* = 13) as well as aseptic loosening (*n* = 12) were found in 8% of all revisions (*n* = 157). Rare causes such as periprosthetic fractures, arthrofibrosis, and defects of the hinge mechanism were summarized as “Others” (13%, *n* = 21).

**Figure 3:**
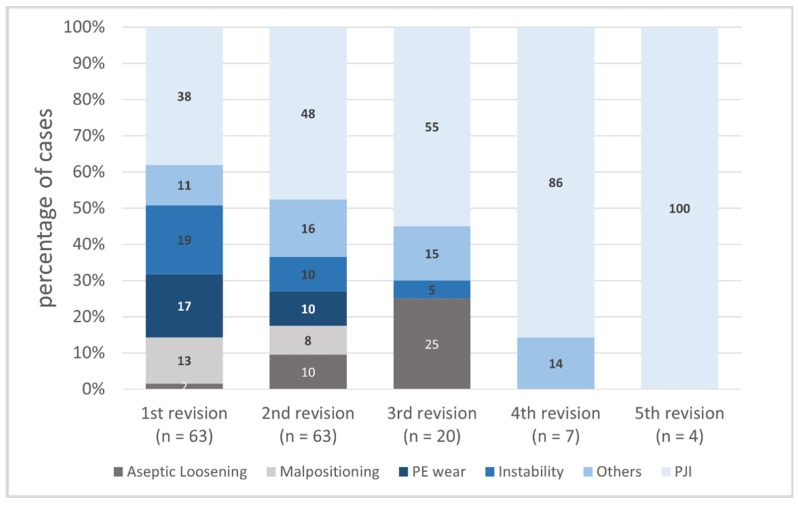
Distribution of revision reasons over the number of changes. PJI = periprosthetic joint infection. PE = polyethylene.

While PJI was the reason for the first revision in 38%, the incidence increased constantly with the number of revisions (48% at second revision, 55% at third revision, 86% at fourth revision, and 100% at fifth revision, *p* = 0.022 Fishers exact test). The incidence of aseptic loosening increased within the first three revisions (2% vs. 10% vs. 25%). The distribution of revision reasons over the number of changes is shown in Figure 3.

Table 1 demonstrates revision indications depending on the cause of the previously performed surgery. In case PJI was treated, the reason for re-revision was in 36.6% again PJI. Overall PJI was in 54.8% the reasons of re-revision followed by “Others” (14%) and aseptic loosening (11.8%). Here, the total number of interventions corresponded to 93 since only the interventions with a follow-up intervention were included in the evaluation.

### 3.4. Characteristics of Septic Revisions

The survival time of the TKA is shortened with an increasing number of revision surgeries (*p* = 0.003, mixed model univariate test). The TKA survival between primary TKA and first revision (*n* = 63) was 53 ± 60 month, between the first and second revision (*n* = 63) 29 ± 32 months, between the second and third revision (*n* = 63) 49 ± 38 months, between the third and fourth revision (*n* = 21) 37 ± 32 months, between the fourth and fifth revision (*n* = 7) 25 ± 25 months, and between the fifth revision and follow-up (*n* = 4) 23 ± 15 months. The comparison of average survivorship between overall septic and aseptic revision indications showed no significant difference. Periprosthetic joint infections showed an overall survivorship of 35.2 ± 5.3 months, aseptic loosening 40.2 ± 12.8 months, instability 39.3 ± 10.3 months, malpositioning 30.8 ± 12.3 months, polyethylene (PE) wear 40.6 ± 11.3 months, and others 46.6 ± 9.5 months averaged over all revision surgeries (*p* = 0.906, mixed model univariate test). Table 2 gives a detailed overview about the survivorship of defined revision indications (*n* = 82) compared to septic (*n* = 75) revisions. There was no significant difference in survivorship of revision comparing initial septic and aseptic revisions (*p* = 0.902; mixed model univariate test).

If the first revision was due to periprosthetic infection (*n* = 24, 38%), patients showed on average two more septic revisions at follow-up than patients with an aseptic first revision indication (*p* < 0.001, Mann–Whitney Test). However, the overall number of revision procedures did not differ significantly in case of aseptic and septic first revisions (PJI 2.6 ± 1.1 vs. aseptic revision 2.4 ± 0.7, *p* = 0.741, Mann–Whitney Test).

Out of 63 total patients, 49 (77.7%) underwent at least one revision due to PJI. Twenty-one (42.5%) of these patients underwent another re-revision due to PJI at follow-up. Overall, 75 of the 157 (47.8%) cases studied were treated for periprosthetic infections. Analyzing those revisions performed due to PJI (*n* = 75), 24% (*n* = 18) were treated by DAIR strategy, 8% (*n* = 6) by one-stage revision, and 64% (*n* = 48) by multiple-stage revision. Four percent (*n* = 3) were identified as PJI after a change of only one component (femoral or tibial). As this treatment does not fit the above-mentioned algorithm, those three cases were excluded from further analysis; 45.8% (*n* = 33) of the remaining 72 cases needed further revision due to reoccurring/persisting PJI.

### 3.5. Impact of Patients’ Characteristics on Implant Survivorship and Number of Revisions

#### 3.5.1. Survivorship

There were no significant differences in overall survivorship of revision TKA depending on gender and BMI (Table 2). In contrast, age influenced TKA survivorship. Younger patients (age < 65) showed significantly longer TKA survivorship than patients aged ≥ 65 years (+25.9 months, *p* = 0.001, mixed model). Table 3 shows the complete results.

#### 3.5.2. Number of Revisions

Whereas gender and age showed no significant impact on number of TKA revisions, patients demonstrating a BMI > 30 showed a significantly higher number of revisions at follow-up. When only evaluating septic revisions, males showed significantly more septic revisions than females (0.9 vs. 1.5, *p* = 0.038, Mann–Whitney Test). Table 4 shows the complete results.

### 3.6. Type of Implant

**Figure 4:**
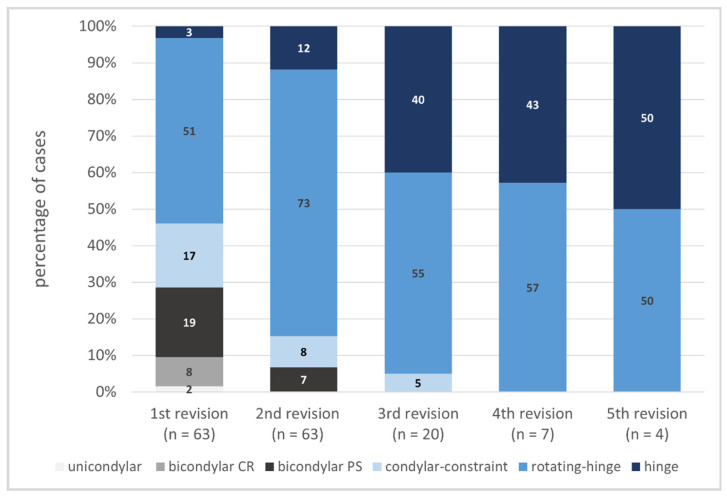
Types of implants used in every revision surgery. There is an increasing percentage of constrained implants with every revision performed. After performing three revisions, nothing but hinge knees were used. CR = cruciate-retaining, PS = posterior stabilized.

Regarding the prosthesis design used, the analysis showed the following distribution pattern: bicondylar design in 26.9% (*n* = 17) (19% posterior stabilized design, 7.9% cruciate retaining design), condylar-constraint (CC) design in 17.5% (*n* = 11), and rotating hinge design in 50.8% (*n* = 32) at first revision surgery. While the implantation of bicondylar and CC designs decreased with every revision, the use of the hinge design increased (Figure 4, *p* < 0.001 Monte Carlo). In 94% (N = 147) of all 157 revisions, a cemented technique was chosen, whereas in 5% (N = 8) cementless stems were used, and in 1% (N = 2) a hybrid technique was seen.

## 4. Discussion

This study is the first monocentric evaluation analyzing failure mechanisms of exclusively multiple TKA revision surgeries. The monocentric inclusion design negates the known influences and limitations of existing registry data. The data analysis is based on identical clinical and therapeutic algorithms, thereby reducing the known register data bias. Focused on the main findings of the study, the proportion of periprosthetic infections increases with the frequency of revision surgeries, and the intervals between revision interventions shorten significantly as the number of surgeries increases.

The predominance of periprosthetic infections in the sense of persistent or new-onset infectious events implies that despite the constant developments in the treatment of PJI, there is a certain percentage of failures in septic treatment strategy. Possible reasons as well as the identification of risk factors are the current topics of research. It is hypothesized that the longer surgical time for complex revision surgery usually presented comorbidities and the averaged older patient population may increase the risk of (re-)infection [22,23,24,25,26,27]. Furthermore, multiple TKA revisions lead to an increase of capsular fibrous tissue, which has lately been shown to have dysfunctional angiogenesis, which might result in an impaired bloody supply [28]. The latter results in worse oxygen load of the vulnerable tissue and might also lead to a reduced antibiotic level in the infected knee joint. Therefore, less antibiotic concentration can reach the knee joint, favoring persistence of PJI. This problem might be addressed by the use of antibiotic loaded spacers in septic revisions providing a high local concentration of antibiotics [29,30]. In contrast, the high thermal response (approximately 46 °C) achieved by using polymethyl-methacrylate (PMMA) for implant fixation or the creation of static spacers in bony defect situations is accompanied with a negative affection of the surrounding soft tissue [31]. All these risk factors are likely to inhibit soft tissue as well as bone quality and, therefore, favor PJI persistence or reoccurrence in multiple revision situations. Besides patient-specific risk factors, inadequate antibiotic or surgical treatment has been shown to favor PJI persistence [32]. Additionally, undiagnosed periprosthetic infections (three cases presented in our study population), certainly represent a considerable risk for persistent or questionable new infections with a high impact on patient outcome. New diagnostic tools such as alpha defensin, immunosorbent assays, cytokine analysis by multiplex protein microarray, S-PECAM, or D-lactat might provide more reliable results [33,34,35,36].

In addition to periprosthetic infections, the study results also show a trend toward increasing revision with aseptic loosening within the first three revision surgeries (2% to 25%). This might be a consequence of decreasing bone quality and reduced amounts of bone marrow as a base for cement fixation with an ongoing number of TKA revisions. As PMMA works by form-closed connection or forced-fit closure [31], the existence of cancellous bone is crucial for adequate implant fixation. The less cancellous bone that is left, e.g., due to radical septic debridement, the less surface there is for interlocking. This might result in a higher incidence of aseptic loosening with each subsequent revision, especially after previous septic revisions with radical debridement [37]. Additionally, the rising incidence of aseptic loosening might be favored due to the increasing use of constraint implant designs with each revision [38]. Consequently, the use of cementless sleeves and stems might be indicated in multiple revision situations regarding the increasing challenge of implant fixation with every revision performed. In our patient population, cemented revision stems have been used in most cases (94%), so in this respect, no confirmation or refutation of the benefit of cementless revision stems can be made.

Instability plays only a minor role in re-revisions. This can be explained by the increasing use of higher constrained implants in re-revisions since the radical nature of revision surgery means that the original anatomy no longer provides sufficient stability.

Although malpositioning of the prosthesis leads to a significantly earlier failure rate according to the available study results [5], the overall incidence of incorrect positioning of the prosthesis is negligible as a reason for multi-revisions. This might be explained by the increasing use of rotating hinge designs with every revision performed, known to be more forgiving concerning malrotation.

Related to patient characteristics, we defined three items (age, sex, and body mass index) as we expected an impact regarding outcome. Only obesity (BMI > 30) was shown to increase the overall number of revisions significantly and showed a trend to increase the number of especially septic revisions. These findings are in line with those of Kerkhoffs et al. and Sisko et al., identifying obesity as a risk factor for TKA (re-)revision [39,40]. Reasons might be longer operation time and reduced oxygen inhibiting proper wound healing [41,42].

The current study has several limitations. Despite a large primary patient population, the restriction to at least two prosthesis revisions and complete documentation greatly reduces the population to be evaluated. Results can therefore not be generalized. The observed drop in survivorship between the first and second revisions (average 29 months) is biased by the chosen study design including only patients with at least two TKA revisions. This means that a reference to the next intervention or final follow-up can only be made from the second revision onward. Another limitation represents the possible systematic impact especially regarding primary TKA performed at different hospitals caused by multiple prosthetic designs and the development of surgical techniques. There might also be a possible impact of reinfection rate due to chosen surgical technique (one-stage vs- two-stage revision). However, current literature shows no inferiority of the one-stage revision if patients with complicating risk factors are excluded [43,44]. By now, prospective randomized trials are started to evaluate a possible bias but data is still missing [45]. However, as only 8% of all septic (re-revisions) were performed as a one-stage revision, a major bias on our data seems neglectable. The incidence of aseptic loosening may be subject to bias due to the predominant use of constrained prosthesis types with a demonstrated higher risk of loosening.

## 5. Conclusions

The probability of survival of the implanted knee arthroplasty is significantly reduced with each subsequent revision. Periprosthetic infection is the main cause of revision and implies that diagnostic algorithms as well as surgical techniques need to be optimized. Overall, faultless implantation of the first knee arthroplasty is all the more to be demanded, since the results underpin the increasingly poor outcome data of revision operations and should be avoided in any case.

## Figures and Tables

**Table 1:** Reason for re-revision depending on previous revision indication.

		Indication for Following Re-Revision						
		PE Wear	Aseptic Loosening	Instability	PJI	Malposition	Others	Total
**Indication for Revision**	PE wear	2 (2.2%)	2 (2.2%)	1 (1.1%)	5 (5.4%)	1 (1.1%)	1 (1.1%)	12 (12.9%)
	Aseptic loosening	0	2 (2.2%)	1 (1.1%)	2 (2.2%)	0	0	5 (5.4%)
	Instability	1 (1.1%)	2 (2.2%)	1 (1.1%)	4 (4.3%)	0	5 (5.4%)	13 (14%)
	PJI	1 (1.1%)	2 (2.2%)	2 (2.2%)	34 (36.6%)	2 (2.2%)	4 (4.3%)	45 (48.4%)
	Malposition	1 (1.1%)	1 (1.1%)	1 (1.1%)	3 (3.2%)	2 (2.2%)	1 (1.1%)	9 (9.7%)
	Other	1 (1.1%)	2 (2.2%)	1 (1.1%)	3 (3.2%)	0	2 (2.2%)	9 (9.7%)
	total	6 (6.5%)	11 (11.8%)	7 (7.5%)	51 (54.8%)	5 (5.4%)	13 (14%)	93 (100%)

PJI = periprosthetic joint infection, PE = polyethylene.

**Table 2:** Impact of the initial revision reason on average TKA survival (mixed model analysis).

Survivorship Compared between Septic and Aseptic Revision Reasons			
Revision Indication	Average Survivorship [Months]	Significance [*p*]	95% Confidence Interval [Months]
PJI	0 ^a^	-	-
Aseptic loosening	4.9 ± 14.1	0.726	−22.9–32.7
Instability	4.1 ± 12.0	0.732	19.5–27.8
Malpositioning	−4.4 ± 13.8	0.750	−31.6–22.8
PE wear	5.3 ± 12.9	0.680	−20.2–30.8
Others	11.4 ± 11.0	0.303	−10.3–33.2

PJI = periprosthetic joint infection, PE = polyethylene, ^a^—zero as analysis referred to this parameter.

**Table 3:** The impact of demographic parameters on average TKA survival (mixed model analysis).

Estimates of Fixed Effects			
	Average Survival [Months]	Significance [*p*]	95% Confidence Interval [Months]
BMI			
<30 kg/m^3^	11.1 ± 7.5	0.145	−3.9–26.1
≥30 kg/m^3^	0 ^a^		-
Gender			
Female	−2.7 ± 7.7	0.731	−18.1–12.8
Male	0 ^a^		-
Age			
<65 years	25.9 ± 7.5	0.001 *	10.9–41
≥65 years	0 ^a^		

BMI = body mass index, * significant, ^a^—zero as analysis referred to this parameter.

**Table 4:** Impact of demographic parameters on average number of overall and especially septic revisions.

Patient Characteristics on Number of Overall Revisions			
	N [%]	Average Number [N]	Significance [*p*]
BMI			
<30 kg/m^3^	32 (51)	2.2 ± 0.4	0.01 *
≥30 kg/m^3^	31 (49)	2.8 ± 1.0	
Gender			
Female	35 (56)	2.5 ± 0.9	1.00
Male	28 (44)	2.5 ± 0.9	
Age			
<65 years	31 (48)	2.6 ± 0.9	0.268
≥65 years	32 (52)	2.4 ± 0.8	
**Patient Characteristics on Number of Septic Revisions**			
BMI			
<30 kg/m^3^	32 (51)	0.8 ± 0.8	0.052
≥30 kg/m^3^	31 (49)	1.5 ± 1.5	
Gender			
Female	35 (56)	0.9 ±1.2	0.038 *
Male	28 (44)	1.5 ± 1.2	
Age			
<65 years	31 (48)	1.1 ± 1.2	0.537
≥65 years	32 (52)	1.3 ± 1.3	

BMI = body mass index, * significant.
